# Resilience in the Face of Disaster: Prevalence and Longitudinal Course of Mental Disorders following Hurricane Ike

**DOI:** 10.1371/journal.pone.0038964

**Published:** 2012-06-26

**Authors:** Robert H. Pietrzak, Melissa Tracy, Sandro Galea, Dean G. Kilpatrick, Kenneth J. Ruggiero, Jessica L. Hamblen, Steven M. Southwick, Fran H. Norris

**Affiliations:** 1 National Center for Posttraumatic Stress Disorder, Veterans Affairs Connecticut Healthcare System, West Haven, Connecticut, United States of America; 2 Department of Psychiatry, Yale University School of Medicine, New Haven, Connecticut, United States of America; 3 National Center for Disaster Mental Health Research, White River Junction, Vermont, United States of America; 4 Department of Epidemiology, Columbia University School of Public Health, New York, New York, United States of America; 5 Department of Psychiatry and Behavioral Sciences, Medical University of South Carolina, Charleston, South Carolina, United States of America; 6 Ralph H. Johnson Veterans Affairs Medical Center, Charleston, South Carolina, United States of America; 7 National Center for Posttraumatic Stress Disorder, White River Junction Veterans Affairs Medical Center, White River Junction, Vermont, United States of America; 8 Geisel School of Medicine at Dartmouth, Hanover, New Hampshire, United States of America; Institute of Psychiatry at the Federal University of Rio de Janeiro, Brazil

## Abstract

**Objectives:**

Natural disasters may increase risk for a broad range of psychiatric disorders, both in the short- and in the medium-term. We sought to determine the prevalence and longitudinal course of posttraumatic stress disorder (PTSD), generalized anxiety disorder (GAD), panic disorder (PD), depression, and suicidality in the first 18 months after Hurricane Ike.

**Methods:**

Six hundred fifty-eight adults representative of Galveston and Chambers Counties, Texas participated in a random, population-based survey. The initial assessment was conducted 2 to 5 months after Hurricane Ike struck Galveston Bay on September 13, 2008. Follow-up assessments were conducted at 5 to 9 and 14 to 18 months after Hurricane Ike.

**Results:**

Past-month prevalence of any mental disorder (20.6% to 10.9%) and hurricane-related PTSD (6.9% to 2.5%) decreased over time. Past-month prevalence of PTSD related to a non-disaster traumatic event (5.8% to 7.1%), GAD (3.1% to 1.8%), PD (0.8% to 0.7%), depression (5.0% to 5.6%), and suicidality (2.6% to 4.2%) remained relatively stable over time.

**Conclusions:**

PTSD, both due to the hurricane and due to other traumatic events, was the most prevalent psychiatric disorder 2 to 5 months after Hurricane Ike. Prevalence of psychiatric disorders declined rapidly over time, suggesting that the vast majority of individuals exposed to this natural disaster ‘bounced back’ and were resilient to long-term mental health consequences of this large-scale traumatic event.

## Introduction

On September 13, 2008, Hurricane Ike, a strong Category 2 storm, struck the Galveston Bay region of Texas. More than 200,000 people residing in this area were heavily affected by this hurricane, which caused 195 deaths, resulted in $29.6 billion in damage, and prompted the largest search-and-rescue operation in U.S. history and largest evacuation of Texans in state history [1]. In November 2008, we launched the Galveston Bay Recovery Study (GBRS), a longitudinal epidemiologic study of Galveston Bay area households that aimed to characterize trajectories and determinants of post-disaster mental health outcomes. The GBRS, which assessed a broad range of psychiatric morbidities, provided a unique opportunity to examine the prevalence and longitudinal course of a diverse range of mental health outcomes in individuals affected by a large-magnitude natural disaster.

Data from the National Comorbidity Survey-Replication (NCS-R) study of a nationally representative sample of U.S. adults suggest that the one-year population prevalence of mental disorders is approximately 6.7% for depression, 3.5% for PTSD, 3.1% for alcohol abuse, 3.1% for generalized anxiety disorder (GAD), 2.7% for panic disorder (PD), and 1.3% for alcohol dependence [2]. Exposure to a natural disaster may increase risk for some of these disorders [3], [4], [5], [6], [7]. For example, data from the National Comorbidity Survey (NCS) indicated a past-year prevalence of 11.3% for PTSD related to natural disasters that involved fire [8]. More recently, a study of 1,452 adults affected by the 2004 Florida hurricanes found that prevalence of PTSD and depression 6 to 9 months after the hurricanes was comparable to general population estimates from the NCS-R (3.6% and 6.1%, respectively), but that the prevalence of GAD (5.5%) was somewhat higher [9]. Another recent study of 797 adults exposed to a typhoon found a higher prevalence of 9.3% for PD, but comparable prevalence of 5.9% for depression, 2.6% for PTSD, and 2.2% for GAD 2.5 months after the disaster [10]. Results of these studies underscore the importance of assessing a broad range of mental health outcomes following disasters, as the expression of psychopathology may extend beyond PTSD and depression, and differ across samples.

To date, most studies of the mental health consequences of disasters have been hampered by two central limitations. First, the vast majority of these studies have been cross-sectional in nature. Available longitudinal data suggest that symptoms and prevalence of psychiatric conditions peak at the initial assessment, which is typically conducted within a year of a disaster, and that they improve over time [4], [5], [6], [7]. In a review of 34 longitudinal panel studies, Norris and colleagues [6], [7] found that mental health improved over time in 27 (79%) panels, with 4 (12.5%) panels showing no change, 1 (3%) showing increasing symptoms, and 2 (6%) yielding mixed results. The patterns of decline in symptoms and prevalence of psychiatric disorders have been more variable in studies that included three or more assessments, with some studies observing simple linear declines (e.g., [11]) and others an initial decline followed by stabilization (e.g., [12]) or a quadratic pattern of change (e.g., [13]). While these studies provide some insight into the longitudinal patterns of mental health outcomes in disaster-affected individuals, they are limited by the recruitment of relatively small samples, assessment of a restricted range of mental health outcomes (most often PTSD and depression), and infrequent employment of panel designs that extend beyond one follow-up assessment.

A second limitation is that very few large-scale epidemiologic studies have obtained samples that represent the pre-disaster population because of displacement that is common after a major disaster (e.g., [14], [15], [16]). This limitation arises from population out-migration that frequently happens after large-scale disasters and the challenges of relocating persons who have moved from a disaster site by the time a study is launched. Consequently, studies that employ sampling approaches that do not obtain representative samples that reflect the pre-event population of an area affected by a disaster may fail to assess important segments of the population who may relocate after a disaster. Thus, results of such studies may provide biased estimates of the prevalence and longitudinal course of mental disorders following a disaster.

The current study advances the disaster mental health literature in two important ways. First, it is one of the first studies to provide a longitudinal assessment of the population prevalence of a broad range of mental disorders in individuals who were first assessed early after a major natural disaster. Second, it provides an assessment of mental health outcomes in a sample that represents the pre-disaster population of the geographic region affected by a disaster. Specifically, we sought to determine the prevalence and longitudinal course of several mental health outcomes, including PTSD, generalized anxiety disorder (GAD), panic disorder, depression, and suicidality, which are often prevalent following exposure to mass disasters [4], [5], [6], [7], [9], [10], [17], [18], but not commonly assessed simultaneously in the same disaster-affected population. Prevalence of alcohol abuse and dependence were also evaluated at the initial assessment. We focused on these disorders, as they are among the most common in the aftermath of disasters and traumatic events, and represent a more comprehensive set of mental health outcomes than has been examined in prior studies [6], [7], [8], [9], [10], [19], [20].

## Materials and Methods

### The Sample

As part of the Galveston Bay Recovery Study (GBRS), a population-based epidemiologic study of mental health in the aftermath of Hurricane Ike, we recruited adults (18 years or older) who had been living in Galveston and Chambers counties, Texas for at least one month prior to the hurricane, which hit the area on September 13, 2008. The methods used to sample study participants have been described in detail elsewhere [21], [22]. Briefly, we divided the two counties into five strata based on level of hurricane damage and likelihood of greatest distress among residents; 80 area segments comprised of multiple census blocks were then selected across the five strata and all households in these areas were identified through a list of addresses obtained from Experian, a credit report agency that is used by research firms to obtain comprehensive sampling frames, as well as through field listing. We selected 2,263 households for participation in the study, with individual respondents randomly selected from all eligible household members upon initial contact.

A total of 658 individuals completed a baseline (Wave 1) interview between November 7, 2008 and March 24, 2009, approximately two to five months after Hurricane Ike, with a cooperation rate of 0.83 [23]. The first follow-up interview (Wave 2) was conducted an average of three months later, from February 6 to June 29, 2009, approximately five to nine months after Hurricane Ike. 529 of the 658 (80%) baseline respondents participated in this second study wave. The third interview (Wave 3) was conducted on average eight months later, from November 19, 2009 through April 13, 2010, approximately fourteen to eighteen months after Hurricane Ike, with 487 participants (74% of those who participated at baseline, and 85% of those who also participated at Wave 2). Participants who were interviewed in Wave 1 but not in Wave 2 could rejoin the study in Wave 3; Wave 3 included 39 respondents who participated in the baseline survey but not in the Wave 2 survey. In the current study, the final study sample consisted of the individuals with data available at each of the three Waves; analyses of trajectories of mental disorders over time are based on 448 individuals who completed Waves 1, 2, and 3.

Interviews were conducted using a computer-assisted interview system. After the study was described, oral informed consent was obtained from participants. Oral informed consent procedures were employed instead of written informed consent, as the vast majority of interviews (88%) were conducted via telephone (the other 12% were conducted in person). This approach to obtaining informed consent, as well as all study procedures, was approved by the Institutional Review Boards of the University of Michigan, Dartmouth College, and Yale University. The baseline (Wave 1) interview lasted approximately 70 minutes; Wave 2 and 3 interviews took an average of 31 and 39 minutes, respectively.

### Measures

We assessed symptoms of a number of mental disorders at each study wave. At the Wave 1 interview, symptoms in the participant’s lifetime prior to the interview were assessed; additional questions about the timing of symptoms allowed us to distinguish between lifetime cases with onset prior to, or only after, Hurricane Ike. The Wave 2 and 3 interviews assessed symptoms that had occurred since the previous interview. Questions about the recency of symptoms at each wave also allowed us to identify current cases in the month prior to the interview.

### Posttraumatic Stress Disorder

We evaluated participants for symptoms of posttraumatic stress disorder (PTSD) related to Hurricane Ike, using the PTSD Checklist – Civilian Version (PCL-C) to assess Diagnostic and Statistical Manual of Mental Disorders (DSM-IV-TR) criteria B (re-experiencing), C (avoidance/numbing), and D (hyperarousal) symptoms [24], [25], [26], [27], [28]. Respondents were asked to report how much they were bothered by each of 17 symptoms with reference to Hurricane Ike as the traumatic event (criterion A1); responses ranged from one (not at all) to five (extremely). Additional questions assessed feelings of terror and helplessness during Hurricane Ike (criterion A2), timing and duration of symptoms (criterion E), and the degree of impaired functioning or distress related to the symptoms (criterion F). Study participants were identified as having Hurricane Ike-related PTSD if they met all six DSM-IV-TR criteria for PTSD [24], specifically reporting: terror or helplessness during the event; being bothered “moderately” or more by at least one re-experiencing symptom (e.g., repeated, disturbing dreams of Hurricane Ike), at least three avoidance symptoms (e.g., avoiding activities or situations that remind the respondent of Hurricane Ike), and at least two increased arousal symptoms (e.g., feeling jumpy or easily startled); duration of symptoms of at least one month; and significant impairment or distress resulting from symptoms. The PCL-C has demonstrated excellent internal consistency and substantial agreement with diagnoses of PTSD compared to the Structured Clinical Interview for DSM-III-R (SCID) and symptom ratings compared to the Clinician-Administered PTSD Scale (CAPS) [26], [27], [28]. Our PTSD measure incorporates all PTSD DSM-IV-TR criteria rather than relying on a particular PCL cutpoint; validation of this modified measure against the CAPS in recent work suggests that the measure has excellent psychometrics and is highly specific for PTSD [29].

We also assessed lifetime PTSD symptoms with reference to another traumatic event (not Hurricane Ike), using a subset of DSM-IV Criterion A potentially traumatic events [24]. Traumatic events included exposure to other disasters, being robbed or mugged, being in a serious accident, and experiencing the sudden, unexpected death of someone close. Respondents were asked to select the traumatic event other than Hurricane Ike that they considered the “worst”, and reported PTSD symptoms related to that event. During Wave 2 and 3 interviews, we evaluated symptoms of Hurricane Ike-related PTSD experienced since the previous interview, as well as symptoms of non-Ike PTSD related to a new traumatic event that had occurred since the previous interview. Cronbach’s alpha for the PCL-C related to Hurricane Ike was 0.95 at Wave 1, 0.95 at Wave 2, and 0.96 at Wave 3; Cronbach’s alpha for the PCL-C related to a traumatic event other than Ike was 0.95 at Wave 1, 0.90 at Wave 2, and 0.93 at Wave 3.

### Generalized Anxiety Disorder

We used the Generalized Anxiety Disorder seven-item scale (GAD-7) to assess generalized anxiety disorder [30]. Respondents were asked if they had ever had a two-week period during which they were bothered by each of seven symptoms (e.g., “feeling nervous, anxious, or on edge”). If they responded in the affirmative, they were asked how often they were bothered during that time period (1 =  several days, 2 =  more than half the days, 3 =  nearly every day) and whether that occurred in the past month. Total scores on the GAD-7 range from 0 to 21. Cronbach’s alpha for the GAD-7 scale ranged from 0.79 to 0.88 in this sample. We identified generalized anxiety cases as those individuals scoring 10 or greater and reporting that these symptoms seemed to occur together [30]. The GAD-7 has been shown to have excellent internal consistency and test-retest reliability [30]. Additionally, a recent validation of the GAD-7 against the SCID yielded good psychometrics and high specificity [29].

### Depression

We evaluated depression using the Patient Health Questionnaire-9 (PHQ-9) [31], [32], [33], [34]. Respondents were asked to report whether there was ever a two-week period during which they were bothered by each of nine symptoms. They were then asked how often they were bothered (as for the GAD-7, responses ranged from “several days” to “nearly every day”) and whether that occurred in the past month. Total scores on the PHQ-9 range from 0 to 27. Cronbach’s alpha for the PHQ-9 scale ranged from 0.79 to 0.89 in this sample. We identified cases of depression as those individuals meeting all criteria for major depressive disorder or other depressive disorder (a.k.a., “minor” or “subthreshold” depression) [24], [34], [35], by reporting that at least two symptoms occurred “more than half the days” or more frequently during the two-week period (suicidal thoughts were counted if present at all), with one of those symptoms being depressed mood or anhedonia [31]. Respondents also had to report that the symptoms seemed to occur together. The PHQ-9 has been found to have excellent internal consistency, test-retest reliability, and construct validity in primary care settings [32] and in the general population [34]. Additionally, in a recent validation against the SCID, the scoring used in this study demonstrated excellent psychometrics and high specificity [29].

### Panic Disorder, Suicidality, Alcohol Dependence and Abuse

We used the Mini-International Neuropsychiatric Interview (MINI) to assess panic disorder, suicidality, alcohol dependence, and alcohol abuse [36]. The MINI has been shown to be valid and reliable when compared to both the SCID and the Composite International Diagnostic Interview (CIDI) [36], [37], [38].

Cases of panic disorder reported having unexpected spells or attacks during which they suddenly felt anxious, frightened, uncomfortable, or uneasy, that surged to a peak within 10 minutes of starting and occurred on more than one occasion. They also reported that at least one such attack was followed by a month or more of persistent fear of having another attack or worry about the consequences of the attack. Finally, they reported at least four (of thirteen) panic symptoms (e.g., trembling or shaking; shortness of breath or difficulty breathing) during the worst spell they could remember [36].

Suicidality was assessed with questions about suicidal ideation, plans, and attempts. Specifically, we identified cases of suicidality as individuals who reported: (a) thinking they were better off dead or wishing they were dead, (b) wanting to harm themselves, (c) thinking about suicide, (d) having a suicide plan, or (e) attempting suicide.

Alcohol dependence and abuse were assessed only at the Wave 1 interview. We identified cases of alcohol dependence as participants who reported three or more of seven behaviors, reported to have occurred at the same time, reflecting a maladaptive pattern of alcohol use: (1) tolerance (i.e., needing to drink more to get the same effect); (2) withdrawal (i.e., experiencing withdrawal symptoms like shaking hands and sweating when cutting down on alcohol, or drinking to avoid these symptoms); (3) drinking more than intended; (4) unsuccessful efforts to reduce or stop drinking; (5) substantial time spent in obtaining, drinking, or recovering from the effects of alcohol; (6) less time spent in social, occupational, or recreational activities because of drinking; and (7) continued drinking despite knowledge of physical or mental health problems caused by drinking. We identified cases of alcohol abuse as participants who reported one or more behaviors consistent with abuse: (1) problems caused by recurrent intoxication despite responsibilities at home, work, or school; (2) recurrent alcohol use in situations that were physically hazardous (e.g., driving a car, operating machinery); (3) legal problems because of drinking; and (4) continued drinking despite social or interpersonal problems caused by drinking. Furthermore, those meeting lifetime criteria for alcohol dependence were not considered cases of alcohol abuse, in line with DSM-IV criteria [24], [39].

### Sociodemographic Characteristics

During the Wave 1 interview, we collected information on sociodemographic characteristics, including gender, age, race/ethnicity, foreign-born status, educational attainment, household income, and current marital status. We also assessed participant experiences with Hurricane Ike, including exposure to potentially traumatic events during the hurricane (e.g., seeing a dead body, having a family member or close friend killed) and stressors in the aftermath of the hurricane (e.g., being displaced from home, financial loss as a result of the hurricane). Additional questions were asked about post-disaster social support, quality of life and functioning, and use of mental health services.

### Statistical Analysis

First, we calculated the prevalence of each disorder at each study wave, among those who participated in that wave. Specifically, we identified current cases in the month prior to the interview at each of the three waves; at Wave 1, we calculated the “lifetime” prevalence of the disorder up to that time, and we identified “lifetime” cases with onset only after Hurricane Ike; and at Waves 2 and 3, we calculated the prevalence of each disorder for the time period since the last interview, and identified new cases, comprised of study participants meeting criteria for the disorder for the first time at that study wave, with no prior history of the disorder. McNemar tests were used to assess the statistical significance of increases or decreases in the past month prevalence of each disorder over time. Second, we restricted the sample to those who participated in all three waves (n = 448) and created branching diagrams to illustrate disorder trajectories. We followed the trajectories of baseline cases and non-cases of Hurricane Ike-related PTSD, further distinguishing between cases with and without current Ike-related PTSD at the Wave 1 interview. For the other disorders, we followed disorder trajectories among four baseline groups, with Hurricane Ike as a key time reference: (1) those who met criteria for the disorder before and after Ike occurred, (2) those who had the disorder in their lifetime, but not since Ike, (3) those who had the disorder since Ike but not prior to Ike, and (4) those who had no history of the disorder before or since Ike at baseline. Branching diagrams were made in this fashion for GAD, panic disorder, depression, and suicidality. Diagrams were not made for non-Ike-related PTSD because we re-evaluated symptoms based on traumatic events that occurred between waves (rather than for the same event at each wave), or for alcohol abuse and dependence because these disorders were not assessed in the Wave 2 and 3 interviews.

All analyses were weighted to account for unequal probabilities of selection across sampling strata and within households and for nonresponse. An additional post-stratification adjustment was applied to match the sample to the population in Galveston and Chambers counties, according to the 2005–2007 American Community Survey [40]. Samples participating in Waves 2 and 3, and in all three waves, were weighted further to account for attrition. We also conducted multiple imputation for missing values using the Sequential Regression Imputation Method implemented in IVEware [41], [42], creating five imputed datasets. All analyses were conducted using SAS-callable SUDAAN (Version 10.0.1, RTI International, 2009) to properly account for the complex sampling design, weights, and multiple imputation.

## Results

Table 1 reports the distribution of demographic and socioeconomic characteristics in the Wave 1 sample. The majority of study participants was white (63.5%), had more than a high school education (55.0%), reported a household income of at least $40,000 in the year before Hurricane Ike (60.2%), and was married (55.0%). After weighting, sample characteristics matched the population in Galveston and Chamber counties, Texas, according to 2005–2007 estimates from the American Community Survey [40]. The samples that participated in the Wave 2 survey (n = 529), the Wave 3 survey (n = 487), and all 3 surveys (n = 448) had nearly identical characteristics to the Wave 1 sample.

**Table 1:** Baseline demographic and socioeconomic characteristics of the sample, compared to American Community Survey data for Galveston and Chambers counties, Texas.

	Wave 1 sample		American Community Survey, 2005–07	
	n	%	n	%
Total	658		228,392	
**Gender**				
Female	394	51.5	117,665	51.5
Male	264	48.5	110,727	48.5
**Age (years)**				
18–24	56	12.7	28,942	12.7
25–34	105	18.0	41,118	18.0
35–44	108	19.6	44,650	19.5
45–54	121	20.7	47,302	20.7
55–64	117	14.5	33,152	14.5
65+	151	14.6	33,228	14.5
**Race/ethnicity**				
White non-Hispanic	394	63.5	131,489	63.7
Black non-Hispanic	102	13.6	28,207	13.7
Hispanic	126	18.5	38,053	18.4
Other non-Hispanic	36	4.3	8,676	4.2
**Born in the United States**				
No	76	11.9	27,144	11.9
Yes	582	88.1	201,248	88.1
**Educational attainment**				
< High school degree	92	16.0	36,673	16.1
High school degree or equivalent	154	29.0	66,479	29.1
Some college	190	32.8	74,877	32.8
College degree or higher	222	22.2	50,363	22.1
**Household income**				
< $20,000	162	19.2	22,493	19.3
$20,000–$39,999	124	20.6	24,517	21.0
$40,000–$59,999	108	15.4	17,860	15.3
$60,000–$99,999	121	23.9	27,701	23.8
$100,000+	143	20.9	24,021	20.6
**Employed** a				
No	278	37.2	81,782	37.2
Yes	380	62.8	138,353	62.8
**Marital status** b				
Married	310	55.0	112,916	54.5
Unmarried but living with a partner	33	6.4	–	–
Separated	35	5.3	5,927	2.9
Divorced	83	7.6	25,740	12.4
Widowed	75	4.8	14,024	6.8
Never been married	121	20.9	48,689	23.5

a Employed the week before Hurricane Ike on a job for pay; “no” includes retirees and others not in the labor force.

b Marital status in the sample cannot be directly compared to American Community Survey (ACS) data because the category “unmarried but living with a partner” is not included in the ACS.

Prevalence estimates of disorders from all three waves are presented in Table 2. At Wave 1, 8.3% reported symptoms that met criteria for PTSD related to Hurricane Ike, with 6.9% having Ike-related PTSD in the past month. Almost all respondents (88.3%) had experienced at least one traumatic event in their lifetime, other than Hurricane Ike; of those individuals, 15.4% had developed PTSD in relation to the “worst” traumatic event and 5.8% met PTSD criteria in the past month. Suicidality, alcohol abuse, and depression had the highest lifetime prevalence (25.7%, 25.4%, and 23.1%, respectively), whereas panic disorder had the lowest prevalence (3.7% lifetime, 0.8% past month). Overall, 60.1% of individuals reported symptoms consistent with at least one of the disorders in their lifetime; 20.6% had one or more disorders in the month prior to the Wave 1 interview.

**Table 2:** Prevalence of mental health outcomes, by survey wave.

	WAVE 1 (n = 658)		WAVE 2 (n = 529)		WAVE 3 (n = 487)	
	2–6 months post-Ike		5–9 months post-Ike		14–18 months post-Ike	
	N	%	N	%	N	%
**POSTTRAUMATIC STRESS DISORDER (PTSD)**						
Hurricane Ike-related PTSD						
Since Hurricane Ike/last interview	69	8.3	28	2.7	30	3.6
Past month	60	6.9	24	2.1	23	2.5
Since but not prior to last interview	–	–	10	0.9	13	1.4
At least one traumatic event experience (other than Ike)	602	88.3	101	20.5	179	41.6
PTSD from worst traumatic event, among those who had an event						
Lifetime/since last interview	100	15.4	5	3.4	15	10.9
Past month	40	5.8	4	3.1	11	7.1
Since but not prior to Hurricane Ike/last interview	10	1.8	3	2.7	11	7.6
**GENERALIZED ANXIETY DISORDER (GAD)**						
Lifetime/since last interview	78	10.7	20	2.3	21	3.6
Past month	26	3.1	20	2.2	13	1.8
Since but not prior to Hurricane Ike/last interview	4	0.5	12	1.2	11	2.7
**PANIC DISORDER**						
Lifetime/since last interview	28	3.7	5	0.6	7	0.7
Past month	7	0.8	4	0.5	6	0.7
Since but not prior to Hurricane Ike/last interview	0	0.0	3	0.2	5	0.3
**DEPRESSION**						
Lifetime/since last interview	151	23.1	33	5.4	40	7.0
Past month	44	5.0	31	4.8	32	5.6
Since but not prior to Hurricane Ike/last interview	5	0.5	17	3.1	11	2.2
**SUICIDALITY**						
Lifetime/since last interview	156	25.7	13	1.8	24	7.4
Past month	21	2.6	8	1.2	14	4.2
Since but not prior to Hurricane Ike/last interview	2	0.1	4	0.3	9	2.8
**ALCOHOL DEPENDENCE AND ABUSE** *						
Alcohol dependence						
Lifetime	47	11.5	–	–	–	–
Past month	7	2.8	–	–	–	–
Since but not prior to Hurricane Ike/last interview	0	0.0	–	–	–	–
Alcohol abuse						
Lifetime	139	25.4	–	–	–	–
Past month	15	3.8	–	–	–	–
Since but not prior to Hurricane Ike/last interview	0	0.0	–	–	–	–
**ANY DISORDER**						
Lifetime/since last interview	371	60.1	68	9.3	77	15.9
Past month	138	20.6	61	8.3	58	10.9
Since but not prior to Hurricane Ike/last interview	83	10.5	40	5.2	44	10.0

* Alcohol abuse and dependence were not assessed in Waves 2 and 3 *Note*. McNemar tests revealed that the decrease in past-month prevalence Hurricane Ike from

Wave 1 to Wave 2, and from Wave 1 to Wave 3 were statistically significant, both p’s<.05. None of the other changes in prevalence were significant.

Apart from Ike-related PTSD, the proportion of new cases at Wave 1 (i.e., with symptom onset only since Hurricane Ike) was low. PTSD from a traumatic event other than Hurricane Ike had the largest prevalence of new cases (1.8% of those with at least one lifetime traumatic event); there were no new cases of panic disorder, alcohol dependence, or alcohol abuse. Overall, 10.5% of respondents had developed symptoms consistent with one or more disorders only after Hurricane Ike. This mostly reflects those who developed PTSD from the hurricane.

In the follow-up waves, the prevalence of Ike-related PTSD declined, with 2.1% and 2.5% meeting criteria in the past month at Waves 2 and 3, respectively. Depression since the previous interview remained stably prevalent (5.4% in Wave 2, 7.0% in Wave 3) and 7.4% of the Wave 3 sample reported suicidality since the previous interview. The Wave 3 prevalence of PTSD from an event other than Hurricane Ike was also particularly high (10.9% among the 41.6% who experienced a traumatic event between Waves 2 and 3). Panic disorder remained the least prevalent disorder (0.6% in Wave 2 and 0.7% in Wave 3). Overall, 9.3% of the Wave 2 sample met criteria for at least one disorder since the baseline interview and 15.9% of the Wave 3 sample had at least one disorder since Wave 2. Among the 448 individuals who completed all 3 survey waves, 8.9% met criteria for Ike-related PTSD, 8.0% for GAD, 2.2% for panic disorder, 16.7% for depression, and 10.5% for suicidality at some time during the roughly 18-month period after Hurricane Ike. Among the 52.4% of participants who reported at least one traumatic event after Hurricane Ike, 14.1% met criteria for PTSD related to their worst traumatic event other than Ike during this period.

Regarding new cases that developed during the follow-up period, 7.6% of Wave 3 respondents who experienced a traumatic event between the Wave 2 and 3 surveys developed PTSD for the first time. Furthermore, 2.8% and 2.7% of the Wave 3 sample met criteria for suicidality and GAD, respectively, for the first time. Overall, 5.2% of the Wave 2 participants had developed a new disorder since the baseline survey, whereas 10.0% of the Wave 3 participants had developed a new disorder since the Wave 2 survey; this includes a very small percentage who had newly developed Ike-related PTSD.

**Figure 1:**
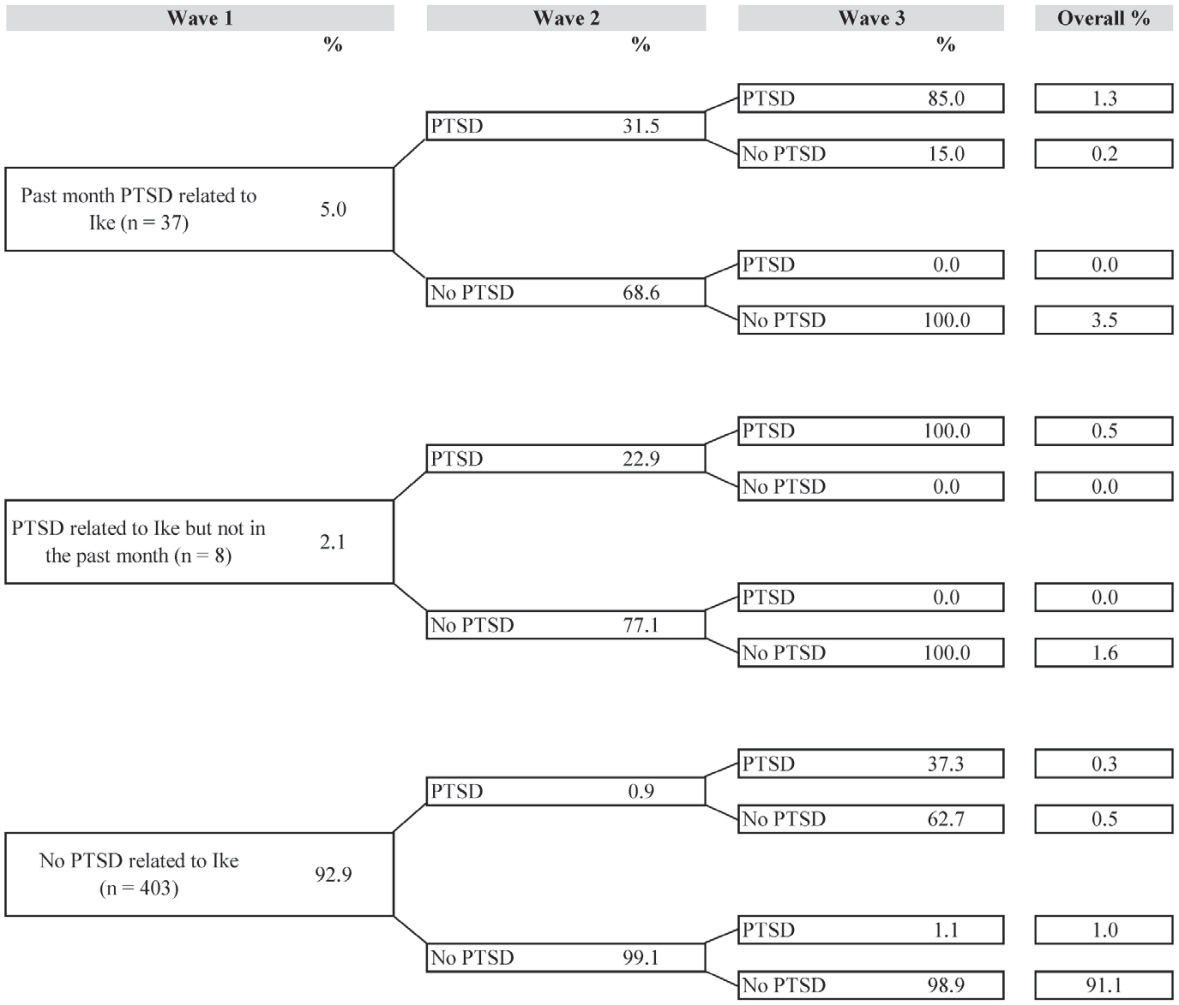
Trajectories of Ike-related PTSD (n = 448).

**Figure 2:**
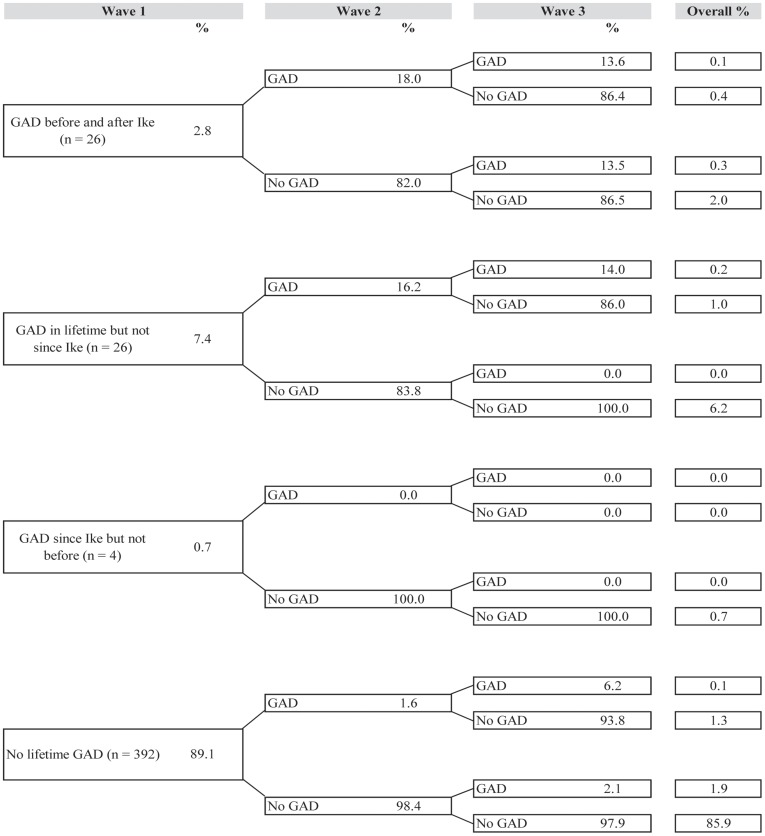
Trajectories of generalized anxiety disorder (n = 448).

**Figure 3:**
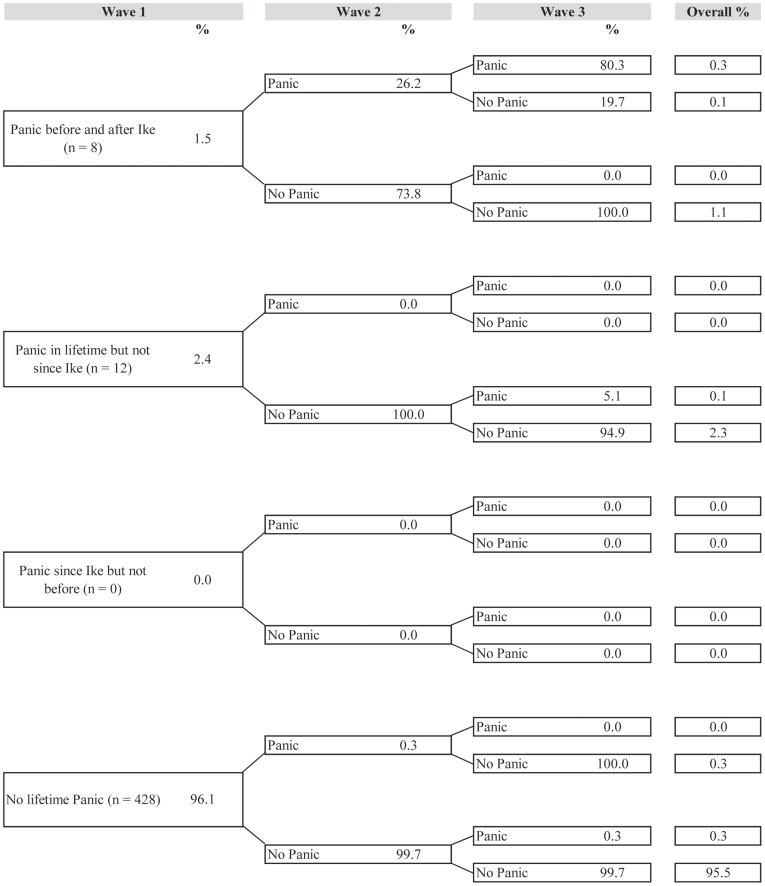
Trajectories of panic disorder (n = 448).

**Figure 4:**
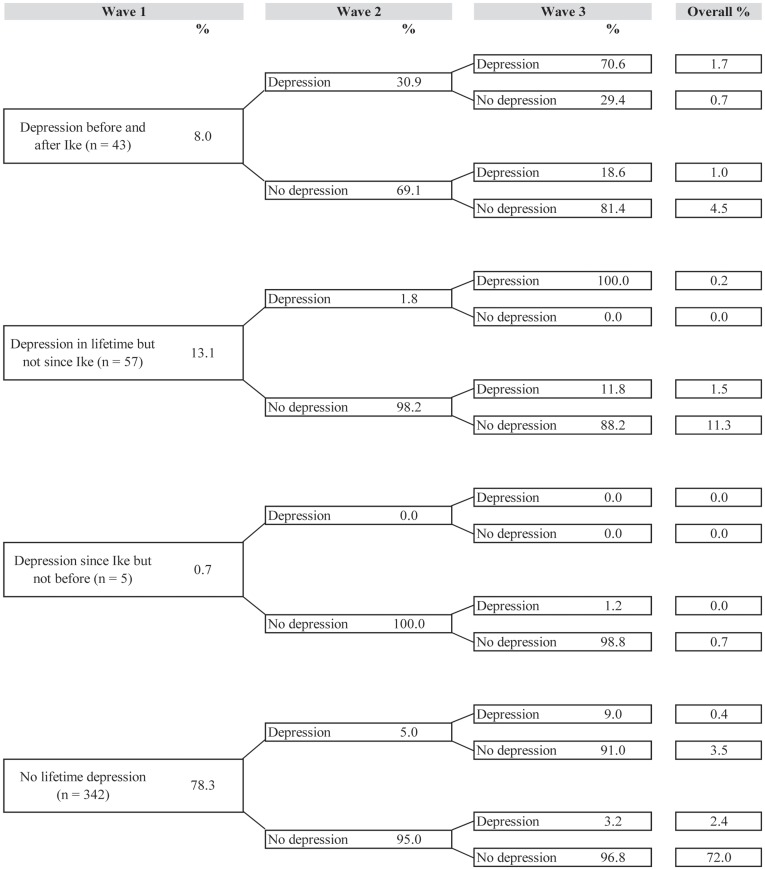
Trajectories of depression (n = 448).

**Figure 5:**
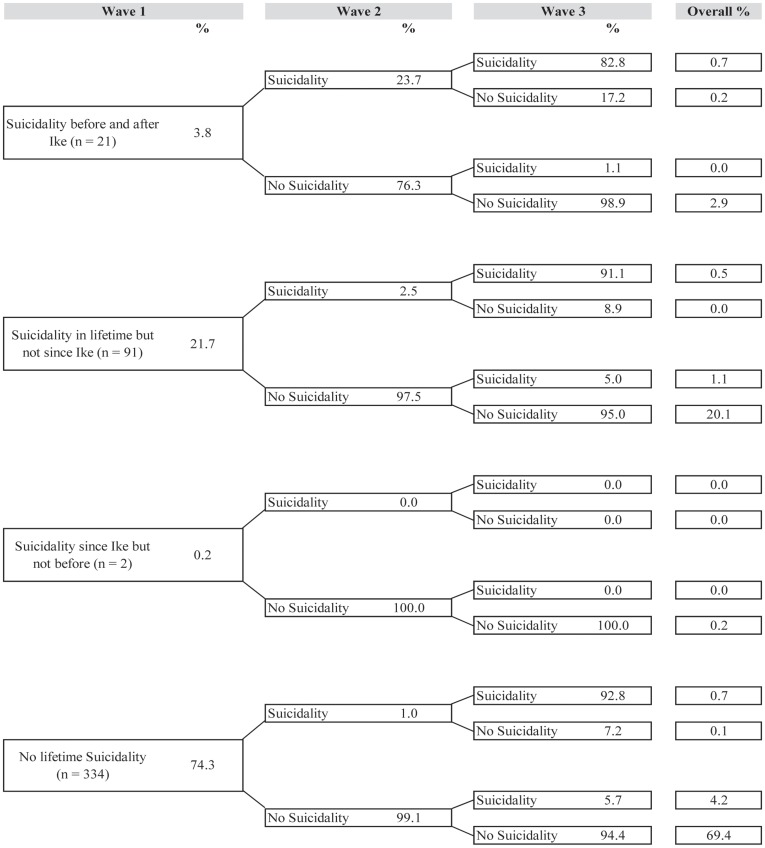
Trajectories of suicidality (n = 448).

Figures 1, 2, 3, 4, and 5 illustrate trajectories of Ike-related PTSD, generalized anxiety disorder, panic disorder, depression, and suicidality among individuals who participated in all three interviews.

Of those who met criteria for Ike-related PTSD at baseline, whether or not they met criteria in the past month, the most common trajectory was resolution of the disorder by Wave 2 with no return by Wave 3 (3.5% and 1.6% of the sample, when starting with Ike-related PTSD in the past month at Wave 1 and Ike-related PTSD since Ike but not in the past month, respectively). Overall, most respondents were resistant to PTSD from Hurricane Ike throughout the study period (91.1%). Only 1.3% of the sample met criteria for Ike-related PTSD at all three waves.

Among those who reported GAD at baseline, most did not continue to meet criteria for the disorder at Waves 2 and 3; for example, none of the 0.7% of the sample who reported initial onset of GAD since Hurricane Ike at the Wave 1 interview continued to meet criteria for the disorder during the follow-up period, and 6.2% of the sample reported lifetime GAD prior to Hurricane Ike but at no time after. Over 85% of the sample was resistant to GAD throughout the study.

Trajectories of panic disorder aside from complete resistance (95.5%) were rare, with 2.3% of the sample meeting criteria for lifetime panic disorder but not at any time since Hurricane Ike and 1.1% of the sample having panic disorder both before and after Hurricane Ike, as reported in Wave 1, but not meeting criteria during the follow-up period.

Similar to the other disorders, most of those who reported depression at baseline did not continue to experience the disorder at Waves 2 and 3. A fairly substantial proportion of the sample (11.3%) met criteria for major or other depressive disorder prior to Hurricane Ike but at no time after; 4.5% of the sample had depressive disorder both before and after Ike at baseline, but did not meet criteria during the follow-up period. Over 70% of the sample never met criteria for depression in their lifetime or during the study period.

Finally, suicidality also showed this same general pattern of trajectories. About twenty percent of the sample experienced suicidality prior to Hurricane Ike but not at any time after, while 2.9% of the sample reported suicidality both before and after Ike at Wave 1 but not during the follow-up period. One exception to the usual pattern was a higher prevalence of new cases of suicidality at Wave 3 than seen for other disorders (4.2% of the sample reported suicidality for the first time at Wave 3). Although the vast majority of respondents was resistant to suicidality at each wave (69.4%), this group was smaller than the resistant trajectory for the other disorders.

## Discussion

The current study is one of the first to provide a longitudinal assessment of the population prevalence of mental disorders in a representative sample of disaster-affected individuals in the early aftermath of a major natural disaster. The rigorous sampling strategy that we employed allowed us to obtain a sample that was representative of the pre-Hurricane Ike population of Galveston and Chambers counties, Texas. Thus, results of this study provide unbiased estimates of the prevalence and trajectories of mental disorders among the pre-disaster population of an area affected by a large-scale disaster. PTSD related to both Hurricane Ike and to other traumatic events, was the most prevalent disorder at the Wave 1 assessment. Longitudinal analyses revealed that past-month prevalence of any disorder decreased by nearly 50% over the 1-year follow-up period, with declining prevalence of Hurricane Ike-related PTSD and relatively stable prevalence of non Ike-related PTSD, GAD, PD, depression, and suicidality.

PTSD, both due to the hurricane and due to other traumatic events, was the most prevalent disorder at the Wave 1 assessment. This finding is consistent with a large body of prior research, which has similarly documented high prevalence of PTSD following disaster exposure [3], [4], [5], [43], [44], [45]. For example, a population-based study of 1,043 individuals affected by Hurricane Katrina found that 16.3% screened positive for probable PTSD [44]. Of note, the prevalence of PTSD documented in the current study is consistent with prior studies that have applied DSM-IV-based diagnostic approaches to operationalizing PTSD (e.g., [46]), but lower than the prevalence of PTSD when assessed using screening criteria (e.g., [47]).

The past-month prevalence of GAD at Wave 1 (3.1%) is consistent with the prevalence of this disorder observed in a recent study of Vietnamese adults affected by a typhoon (2.2%; [10]); however, it is slightly lower than that observed in a study of adults affected by the 2004 Florida hurricanes (5.5%; [9]). The past 14 to 18-month prevalence of GAD (2.7%) was comparable to the 3.1% past 12-month prevalence of this disorder in the general U.S. adult population [2]. The past-month prevalence of panic disorder of 0.8% at Wave 1 was lower than the 9.3% prevalence documented in a study of Vietnamese adults affected by a typhoon [10]. The past 14 to 18-month prevalence of panic disorder (2.2%) in the current sample was comparable to the 2.7% past 12-month prevalence of this disorder in the general U.S. adult population [2]. The past-month prevalence of depression of 5.0% at Wave 1 is consistent with the prevalence of depression observed in other recent studies of adult disaster survivors, which have documented a prevalence of depression ranging from 3.9% to 6.1% (e.g., [9], [10], [48]). The past 14 to 18-month prevalence of depression (16.7%) is higher than the 6.7% past 12-month prevalence of depression in the general U.S. adult population [2]; this difference is likely attributable to our including major depression, as well as ‘minor’ or subthreshold depression in our screening definition. Finally, the past-month prevalence of suicidality of 2.6% at the Wave 1 assessment is consistent with the 2.8% prevalence of suicidal ideation among Hurricane Katrina survivors [45]; the past 14 to 18-month prevalence of suicidality (10.5%) in the current sample is higher than the 2.6% past 12-month prevalence of suicidal ideation in the general U.S. population [2].

Evidence regarding changes in substance use after disasters is mixed [49], [50]. Past-month prevalence of alcohol abuse and dependence, which were evaluated only at the Wave 1 assessment, were 3.8% and 2.8%, respectively. These estimates are largely consistent with recent U.S. adult population-based epidemiologic surveys, which have found past-year prevalences of 3.1% to 4.7% for alcohol abuse and 1.3% to 3.8% for alcohol dependence [2], [51]; in the current study, alcohol abuse and dependence were only assessed at Wave 1 (2 to 5 months after Hurricane Ike), so we were not able to compare the prevalence of these disorders with a comparable timeframe as prior nationally representative surveys (e.g., past 14 to 18 months). Of note, the lifetime prevalence of alcohol abuse in the current sample (25.4%) was higher than the 13.2% to 17.8% lifetime prevalence found in these studies. Differences in demographic characteristics, employment of a non-clinician administered and abbreviated structured measure of alcohol abuse/dependence, and recent exposure to a large magnitude natural disaster likely account for these differences. Longitudinal studies of trajectories of alcohol use and abuse following disaster (e.g., [49], [50], [52]) will be helpful in elucidating the relation between disaster exposure and changes in drinking behavior.

Several explanations may account for the lower prevalence of most disorders observed in the current study compared to prior work after disasters. These include differences the magnitudes of disaster exposure across studies and timeframes of the initial assessments; variability in psychopathology measures and/or employment of more rigorous criteria to ascertain probable diagnoses; and possible cultural differences in the expression of psychopathology. Another possibility is that, as residents of the Galveston Bay area, which is frequently affected by hurricanes, the individuals who participated in the current study may have been “stress inoculated” and better prepared to deal with the consequences of such events [53], [54]. While we could not directly test this possibility, as we did not assess whether respondents were lifelong residents of this region of the country, 47.8% of the sample did report prior exposure to a natural disaster at Wave 1. Thus, it is certainly plausible that the observed resilience to negative mental health outcomes in this sample is, at least in part, attributable to nearly half of the sample having prior experience in recovering from natural disasters. Importantly, given that we employed DSM-IV-based operationalization of all diagnoses, the prevalence of psychiatric disorders documented in this study represent more diagnostically accurate estimates of the burden of mental illness following exposure to a major natural disaster.

We found that past-month prevalence of any disorder decreased by nearly 50% a year after Hurricane Ike, with declining prevalence of Hurricane Ike-related PTSD and GAD, and relatively stable prevalence of depression, PD, and suicidality. These results persisted when analyses were limited to the 448 respondents who completed all three assessments. For example, of the 5% of respondents with Hurricane Ike-related PTSD at Wave 1, 68.6% no longer met criteria for this disorder at Wave 2, and all of these respondents were free of Ike-related PTSD at Wave 3. These findings are consistent with a review of 34 longitudinal panel studies of psychopathology following disasters, which found reductions in mental health symptoms over time in 79% of these studies. As observed in prior studies (e.g., [4], [6], [7], [55], [56], [57], [58], [59], these results suggest that the vast majority of individuals exposed to a major natural disaster are resilient to long-term mental health difficulties. Although the prevalence of some disorders, most notably PTSD in this sample, may be elevated in the first few months after a disaster, these prevalences decline and remain at levels consistent with general population estimates a few months later.

Different patterns of changes in prevalence of psychiatric disorders were evident over the three waves of assessment. Changes in prevalence of Hurricane Ike-related PTSD and GAD were characterized by an initial decline at the Wave 2 assessment followed by stabilization at the Wave 3 assessment. Prevalence of PTSD related to a trauma other than Hurricane Ike, depression, PD, and suicidality, on the other hand, remained stable across assessments; and the prevalence of suicidality increased from Wave 2 to Wave 3. While not statistically significant, numerical prevalence rates suggested a “quadratic” pattern of change in PTSD related to a traumatic event other than Ike and suicidality. This finding is in line with results of a previous study of a population-based sample of Hurricane Katrina survivors, which observed that the prevalence of suicidality increased over time [45]. At present, however, there is insufficient longitudinal research that can shed light on these patterns, nor can we draw conclusive inferences about these results. Nevertheless, these findings underscore both the importance of further longitudinal work in this context and of comprehensive screening for mental health outcomes in disaster-affected individuals, as outcomes that are not commonly assessed following disasters – PTSD related to a non-disaster traumatic event and suicidality – may remain stable and elevated one year following a major natural disaster.

Methodological limitations of this study, which are typical of studies in the disaster research field, also should be noted. First, information regarding most mental health outcomes was assessed via retrospective self-report and a pre-disaster baseline assessment of these outcomes was not obtained. Second, current psychological distress may have influenced reporting of past symptoms, thereby possibly exaggerating reports of severity of these symptoms. Third, although we assessed a broad range of mental disorders, other post-disaster behavioral health problems such as interpersonal violence [60], [61], which have been studied in prior work, were not assessed. Additional prospective studies are needed to evaluate trajectories of such problems in the aftermath of a mass disaster.

Despite these limitations, results of this study provide new insight into the prevalence and longitudinal course of a broad range of mental health outcomes first assessed early after a large-magnitude natural disorder. Results suggest that PTSD related to the disaster and a non-disaster traumatic event are the most prevalent mental disorders 2–5 months following exposure to a major natural disaster, and that while the prevalence of most psychiatric disorders decline over time, some outcomes, such as depression and PTSD related to a non-disaster traumatic event, as well as suicidality, remain stable over time and consistent with population-based estimates. These results underscore the importance of comprehensive screening and monitoring of mental health outcomes in disaster-affected individuals.
